# Management of pregnant women in tertiary maternity hospitals in the Paris area referred to the intensive care unit for acute hypoxaemic respiratory failure related to SARS-CoV-2: which practices for which outcomes?

**DOI:** 10.1186/s13613-024-01313-2

**Published:** 2024-06-18

**Authors:** Frédérique Schortgen, Cecilia Tabra Osorio, Suela Demiri, Cléo Dzogang, Camille Jung, Audrey Lavenu, Edouard Lecarpentier, for the COVADIS-PREG study group

**Affiliations:** 1 Department of Adult Intensive Care, Service de médecine intensive réanimation, Centre Hospitalier Intercommunal de Créteil, 40 avenue de Verdun, 94000 Créteil, France; 2 Department of Obstetrics and Gynaecology, Centre Hospitalier Intercommunal de Créteil, Créteil, France; 3 Research Centre, Centre Hospitalier Intercommunal de Créteil, Créteil, France; 4 Université Paris Est Créteil (UPEC), Créteil, France; 5 IRMAR, Mathematical Research Institute, University of Rennes, Rennes, France; 6 Clinical Investigation Centre, INSERM CIC 1414, University of Rennes, Rennes, France

## Abstract

**Background:**

Evidence for the management of pregnant women with acute hypoxaemic respiratory failure (AHRF) is currently lacking. The likelihood of avoiding intubation and the risks of continuing the pregnancy under invasive ventilation remain undetermined. We report the management and outcome of pregnant women with pneumonia related to SARS-CoV-2 admitted to the ICU of tertiary maternity hospitals of the Paris area.

**Methods:**

We studied a retrospective cohort of pregnant women admitted to 15 ICUs with AHRF related to SARS-CoV-2 defined by the need for O_2_ ≥ 6 L/min, high-flow nasal oxygen (HFNO), non-invasive or invasive ventilation. Trajectories were assessed to determine the need for intubation and the possibility of continuing the pregnancy on invasive ventilation.

**Results:**

One hundred and seven pregnant women, 34 (IQR: 30–38) years old, at a gestational age of 27 (IQR: 25–30) weeks were included. Obesity was present in 37/107. Intubation was required in 47/107 (44%). Intubation rate according to respiratory support was 14/19 (74%) for standard O_2_, 17/36 (47%) for non-invasive ventilation and 16/52 (31%) for HFNO. Factors significantly associated with intubation were pulmonary co-infection: adjusted OR: 3.38 (95% CI 1.31–9.21), HFNO: 0.11 (0.02–0.41) and non-invasive ventilation: 0.20 (0.04–0.80). Forty-six (43%) women were delivered during ICU stay, 39/46 (85%) for maternal pulmonary worsening, 41/46 (89%) at a preterm stage. Fourteen non-intubated women were delivered under regional anaesthesia; 9/14 ultimately required emergency intubation. Four different trajectories were identified: 19 women were delivered within 2 days after ICU admission while not intubated (12 required prolonged intubation), 23 women were delivered within 2 days after intubation, in 11 intubated women pregnancy was continued allowing delivery after ICU discharge in 8/11, 54 women were never intubated (53 were delivered after discharge). Timing of delivery after intubation was mainly dictated by gestational age. One maternal death and one foetal death were recorded.

**Conclusion:**

In pregnant women with AHRF related to SARS-CoV-2, HFNO and non-invasive mechanical ventilation were associated with a reduced rate of intubation, while pulmonary co-infection was associated with an increased rate. Pregnancy was continued on invasive mechanical ventilation in one-third of intubated women.

*Study registration* retrospectively registered in ClinicalTrials (NCT05193526).

**Supplementary Information:**

The online version contains supplementary material available at 10.1186/s13613-024-01313-2.

## Introduction

SARS-CoV-2 infection significantly increases the risk of ICU admission and/or preterm birth [1]. In 2022, a systematic review reported thousands of women admitted to the ICU with COVID [2]. Among the 176 686 pregnant women with COVID included in 119 studies, 1.7% were admitted to the ICU, mainly during the period when the delta variant predominated [2, 3]. Studies specifically designed to report experiences in managing pregnant women admitted to the ICU with severe COVID are, however, limited, and there is a need to identify best practices in respiratory and obstetrical management.

Whether general guidelines for respiratory support and ventilatory settings can be applied to these women remains unknown [4–6]. Positive pressure ventilation using a face mask may predispose pregnant women to a higher risk of gastric aspiration [6]. Prone positioning is technically challenging and there is little information on its effects on maternal and foetal perfusion [7].

Whereas placental dysfunction is an indication for childbirth or termination of pregnancy to improve the mother’s condition, such reasoning is debatable when the maternal complication is not pregnancy-related [8]. Case series including women intubated for SARS-CoV-2 pneumonia report no effect or interindividual variability in post-delivery changes in respiratory parameters associated with the mortality of patients with ARDS, i.e. plateau and driving pressures [9–11]. Provision of neuraxial anaesthesia was suggested early in the pandemic to avoid intubation and aerosolization of viral particles [12]. However, the feasibility of neuraxial anaesthesia in patients with acute hypoxaemic respiratory failure (AHRF) has never been assessed.

The aim of this study was to describe practices in managing pregnant women suffering from AHRF related to SARS-CoV-2 pneumonia admitted to the ICU of referral maternity hospitals in the Paris area. This study focused on three main aspects of management. Firstly, we report the feasibility of neuraxial anaesthesia for delivery avoiding intubation. Secondly, factors associated with intubation were studied, mainly the choice of oxygenation support. Thirdly, we tried to identify different trajectories according to the timing of delivery with the possibility of continuing the pregnancy after intubation.

## Methods

This retrospective multicentre cohort study was performed in tertiary maternity hospitals with an available adult ICU and/or maternity hospitals with an available adult ICU with ECMO in the Paris area. Fifteen of the 16 maternity hospitals fulfilling these criteria agreed to participate. The study was approved by the ethics committee of the Centre Hospitalier Intercommunal de Créteil (no. 2021–10-03). According to French law, informed consent was waived, but the patients included and alive were informed in writing about the study. The study complied with the Strengthening the Reporting of Observational Studies in Epidemiology (STROBE) statement guidelines (Supplemental appendix).

From February 2020 to September 2021 (i.e. the first three waves of pandemic), we included all pregnant women over 18 years of age at a gestational age > 14 weeks consecutively admitted to the ICU for AHRF related to SARS-CoV-2 pneumonia proven by a positive real-time reverse transcriptase-polymerase chain reaction assay. AHRF was defined by the need for standard O_2_ ≥ 6 L/min and/or high-flow nasal oxygen (HFNO), and/or non-invasive mechanical ventilation (NIV) and/or invasive mechanical ventilation during ICU stay. Women referred to the ICU requiring invasive mechanical ventilation within 24 h post-delivery were also to be included. We defined AHRF according to the oxygen criteria indicating ICU admission of pregnant women with COVID provided by the regional health agency of the Paris area during the pandemic. In the absence of a consensual definition of hypoxaemia, we believe that this was a pragmatic criterion common to all participating centres located in the same region. The exclusion criterion was an unavailable medical chart.

An electronic case report form was specifically developed for the study. Dedicated research personnel collected data from the ICU and anaesthesia and obstetrical medical records. Data collection and monitoring were centralized at the research centre of the Centre Hospitalier Intercommunal de Créteil. The validity of data extraction from medical files was reviewed by FS and EL. Patient characteristics were recorded at the hospital and at ICU admission. Obesity was defined in reference to the weight before pregnancy. In patients on standard O_2_, inspired oxygen fraction (FiO_2_) was calculated as follows: FiO_2_ = (oxygen flow × 3) + 21 [13]. Respiratory support in the ICU was standard O_2_, HFNO, NIV at either one (continuous positive airway pressure) or two (bi-level positive airway pressure) levels of pressure and invasive mechanical ventilation. Because different respiratory supports can be used in the same patient, concomitantly or over time, before intubation, the invasiveness of respiratory devices was ranked assuming that standard O_2_ is less invasive than HFNO and that HFNO is less invasive than NIV. Patients in whom more than one non-invasive respiratory support was used were classified in the most invasive group. NIV started because of HFNO failure was designated as rescue therapy. In patients requiring intubation, the use of non-invasive respiratory support was recorded before intubation only. Pulmonary bacterial co-infection was defined by the need for antibiotics. Co-infection was classified as documented if a microorganism was identified in pulmonary secretions by standard culture or a multiplex PCR test. In women on invasive mechanical ventilation, only positive cultures within 48 h after intubation were considered.

Maternal trajectories were described according to the time of delivery in reference to ICU admission and intubation. We considered both ICU admission and intubation as potential markers of maternal worsening triggering the decision to deliver. Timing of delivery was arbitrarily defined as early when it occurred within 2 days of ICU admission or of intubation. Foetal monitoring was performed in all participating ICUs according to local practices by ultrasound or foetal heart rate monitoring according to the term of pregnancy.

Respiratory outcome was the need for intubation. This was the primary and only analytic endpoint. Obstetric outcomes were the proportion of women requiring delivery, preterm delivery, complications related to ICU stay and hospital mortality. Neonatal and foetal complications included death, NICU admission and preterm birth: extremely (< 28 0/7 w) very (28 0/7–31 6/7 w) and moderately (32 0/7–35 6/7 w) preterm.

### Statistical analysis

Results are presented as median and interquartile 25^th^–75th range for continuous variables and number (percentage) for categorical variables. Comparisons of intubated and non-intubated patients were performed by means of Student’s t-test or Mann–Whitney tests for continuous variables according to their distribution. Between-group comparisons of the 3 classes of non-invasive respiratory supports and the 4 different trajectories of delivery were performed using the Kruskal–Wallis test. Categorical variables were compared by Chi-square or Fisher exact tests. Stepwise logistic regression was used for building the best logistic regression model. Factors associated with intubation with a p < 0.2 in univariate analysis were entered in the stepwise selection. Time to intubation curves according to respiratory support were constructed using Kaplan–Meier method with multivariate Cox regression up to ICU discharge. No women died without intubation. No imputation was done for missing data. The SAPS-2 score was not included in the variable selection because more points are assigned for PaO_2_:FiO_2_ in intubated patients within 24 h after admission. Because of the retrospective design, it was not possible to record the exact time of intubation and calculate the SAPS-2 within the preceding 24 h. Analyses were performed with R version 4.2.1 [14]. All tests were two-sided, and a p value < 0.05 was considered statistically significant. When a post hoc two-by-two comparison was performed, a Bonferroni correction was applied with a significant p value < 0.025.

## Results

### Population characteristics

**Fig. 1:**
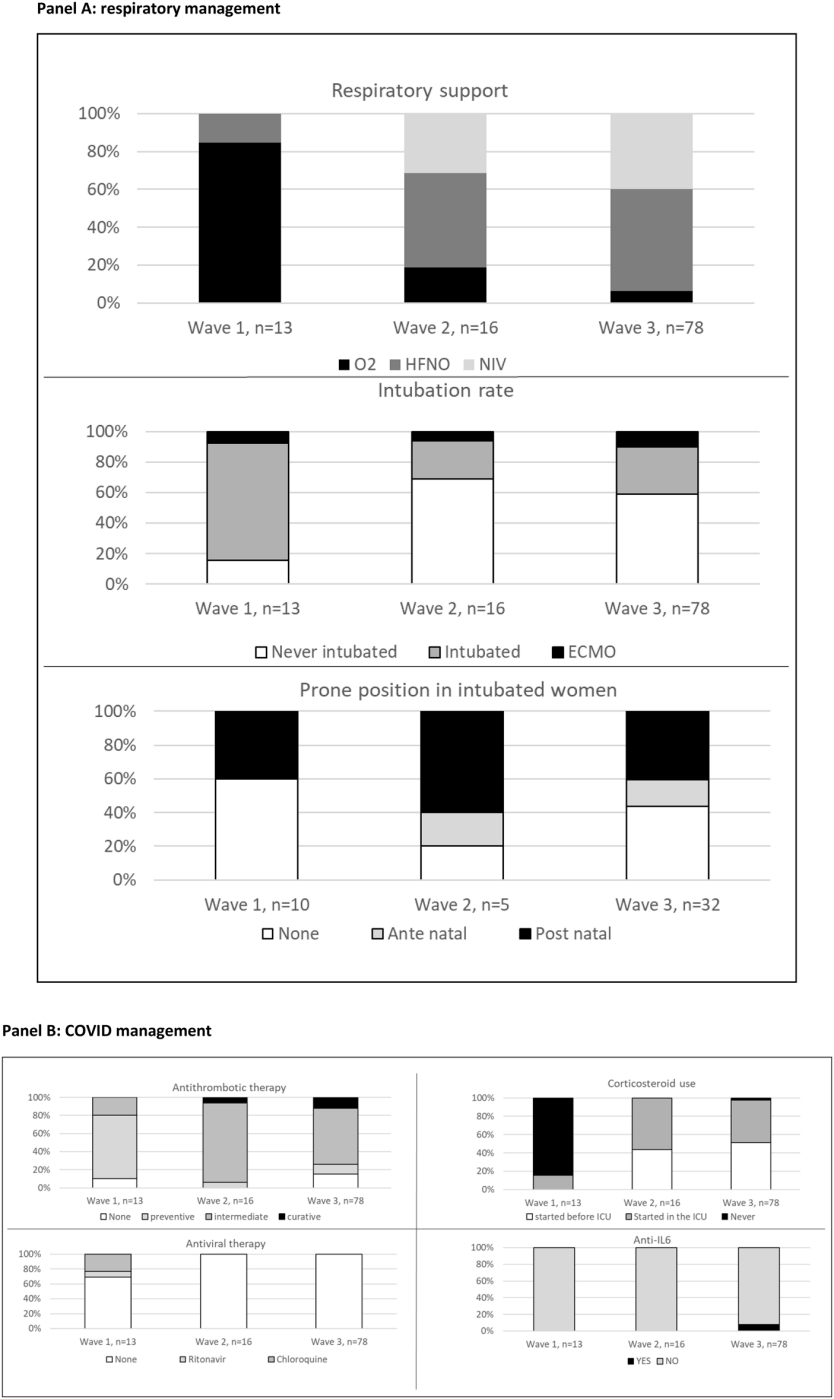
Evolution of respiratory and COVID management over the study period. *HFNO* high-flow nasal oxygen, *NIV* non-invasive mechanical ventilation. **A** respiratory management. **B** COVID management

Of the 107 women included, all were pregnant at ICU admission (see flow chart in fig S1), and 27 were admitted after referral to a level 3 maternity hospital. Seventy-eight of 107 women (73%) were admitted during the period when the delta variant predominated in France (Figure S2). None of the women was vaccinated against COVID. In France, vaccination of pregnant women was recommended in March 2021 for the second trimester and in July 2021 for the first trimester, so that few of the patients included could had been vaccinated. Patients were 34 (IQR: 30–38) years old, 37 (37%) were obese, and 26 (24%) had diabetes mellitus (Table 1). Median gestational age at hospital admission was 27 (25–30) weeks; 54 (50%) patients were at less than 28 weeks of gestation, and 27 (25%) were primiparous. The interval between onset of the first symptoms and hospital admission was 7 (IQR: 4–8) days. Thirty-three women (31%) experienced pulmonary bacterial co-infection, 17 were documented with 6 *Staphylococcus aureus*, 5 *Haemophilus*, 2 *Streptococcus*, and 4 multi-microbial infections. A CT-scan was performed in 87 (81%) patients, 27/87 (31%) showing an extent of at least 50%. The median PaO_2_:FiO_2_ ratio at ICU admission was 165 (130–208) mmHg. Overall, 95/107 (89%) patients received corticosteroids for pneumonia, started before ICU admission in 51/107 (48%). Corticosteroids were more frequently started before ICU admission in the second and third waves (Fig. 1). Dexamethasone was used in 85/95 (89%) and prednisone in 10/95 (11%) women for a median duration of 10 (10–10) days. Antiviral therapy was used in 6 patients, all during the first wave (Fig. 1). Anti-IL6 was used in 6 patients (Fig. 1). Intermediate preventive antithrombotic therapy was prevalent in the second and third waves (Fig. 1).

**Table 1:** Characteristics of the 107 women included according to the need for intubation

	Available in n	All *n* = 107	Intubated *n* = 47	Not intubated *n* = 60	p
Age, y	107	34 (30–38)	35 (31–37)	33 (30–39)	0.48
Hypertension, *n* (%)	107	3 (3)	2 (4)	1 (2)	0.58
Diabetes, *n* (%)	107	26 (24)	13 (28)	13 (22)	0.47
Body mass index, kg/m^2^	99	28.2 (23.3–31.3)	28.4 (23.1–31.0)	28.1 (24.5–31.6)	0.87
Obesity, *n* (%)	99	37 (37)	17 (36)	20 (33)	0.76
Pneumonia characteristics					
Time from 1st symptoms to hospital admission, d	105	7 (4–8)	6 (3–8)	7 (5–8)	0.11
CT scan, *n* (%) Extent ≥ 50%, *n* (%)	87 82	87 (81) 27 (33)	39 (80) 11 (30)	48 (83) 16 (35)	0.68 0.99
Pulmonary co-infection, *n* (%)	107	33 (31)	21 (45)	12 (20)	< 0.01
Obstetric characteristics					
Primiparity, *n* (%)	107	27 (25)	12 (26)	15 (25)	0.95
Twin pregnancy, *n* (%)	107	1 (1)	1 (2)	0	0.44
Gestational age on admission, w Gestational age < 28 weeks, *n* (%)	107	27 (25–30) 54 (50)	28 (26–31) 19 (40)	27 (24–30) 35 (58)	0.13 0.07
Characteristics at ICU admission					
Time from hospital admission, d	107	2 (1–3)	2 (1–3)	2 (1–3)	0.40
SAPS II score*, points	85	21 (17–27)	24 (19–31)	19 (15–24)	0.009
Steroids (including betamethasone) started before ICU admission, *n* (%)	107	51 (48)	19 (40)	32 (53)	0.19
Admission from:	107				0.18
Emergency room		18 (17)	4 (8)	14 (23)	
Prehospital mobile ICU		2 (1)	1 (2)	1 (2)	
Maternity		52 (49)	25 (53)	27 (45)	
Medical ward		12 (11)	4 (9)	8 (13)	
Inter-hospital obstetric referral		23 (2)	13 (28)	10 (17)	
Oxygenation parameters					
PaO_2_:FiO_2_ ratio mmHg	99	165 (130–208)	160 (112–219)	166 (134–200)	0.56
PaO_2_:FiO_2_ < 100 mmHg, *n* (%)	99	7 (7)	6 (13)	1 (2)	0.02
pH	66	7.44 (7.40–7.46)	7.43 (7.41–7.46)	7.44 (7.39–7.47)	0.83
PaCO_2_, mmHg	66	32 (28–34)	31 (26–34)	32 (29–34)	0.75
Respiratory rate, b/min	80	30 (25–37)	30 (26–37)	30 (25–37)	0.74
Vasopressor	107	1 (1)	1	0	0.43
Respiratory support, *n* (%)					
	107				< 0.01
O_2_ only (reference)		19 (18)	14 (30)	5 (8)	
HFNO		52 (48)	16 (35)	36 (60)	
NIV		36 (34)	17 (36)	19 (32)	
Prone positioning, n %	107	29 (27)	25 (53)	4 (7)	< 0.001
Started before delivery		9 (9)	6 (13)	3 (5)	
ECMO, *n* %	107	10 (9)	10 (21)	–	

*HFNO* high-flow nasal oxygen, *NIV* non-invasive ventilation, *ECMO* extracorporeal membrane oxygenation. *Calculated within the 24 h from ICU admission.

### Respiratory management

Non-invasive respiratory supports received during ICU stay were standard O_2_ in 19/107 (18%) patients, HFNO in 52/107 (48%), NIV in 36/107 (33%) combined with HFNO in 33/36 and with O_2_ in 3/36 (Table 1). Among the 80 women in whom information was available, HFNO was started before ICU admission in one, on the day of ICU admission in 40 and on day 2 in 4 patients. NIV was started before ICU admission in one, on the day of ICU admission in 25 patients, and on day 2 in 3 patients. NIV was used as rescue therapy in 3 patients, one of whom required intubation. The use of both HFNO and NIV increased over time and was prevalent in the second and third waves (Fig. 1).

Overall, 47 women required intubation in a median delay of 4 (2–5) days after hospital admission, and 38 (81%) were intubated within 2 days after ICU admission (see figure S2 panel B for intubation rate by centre). Two additional patients were intubated for caesarean section only and ventilated for less than 1 day; these 2 patients were considered as non-intubated in the analysis.

Prone position was applied in 29 (27%) women, 4 in awake patients and 25 after intubation. Nine women were prone-positioned during ongoing pregnancy (Table 1 & figure S3). Thirty-four of the 47 intubated patients received neuromuscular blocking agents and 4 received nitric oxide, all four after delivery. Barotrauma occurred in 4 patients, all after delivery and on invasive mechanical ventilation. Ten patients required ECMO, all were veno-venous, 2 before and 8 after delivery. Median duration of invasive mechanical ventilation was 9 (4–21) days. Five patients were tracheostomized.

### Intubation rate

**Fig. 2:**
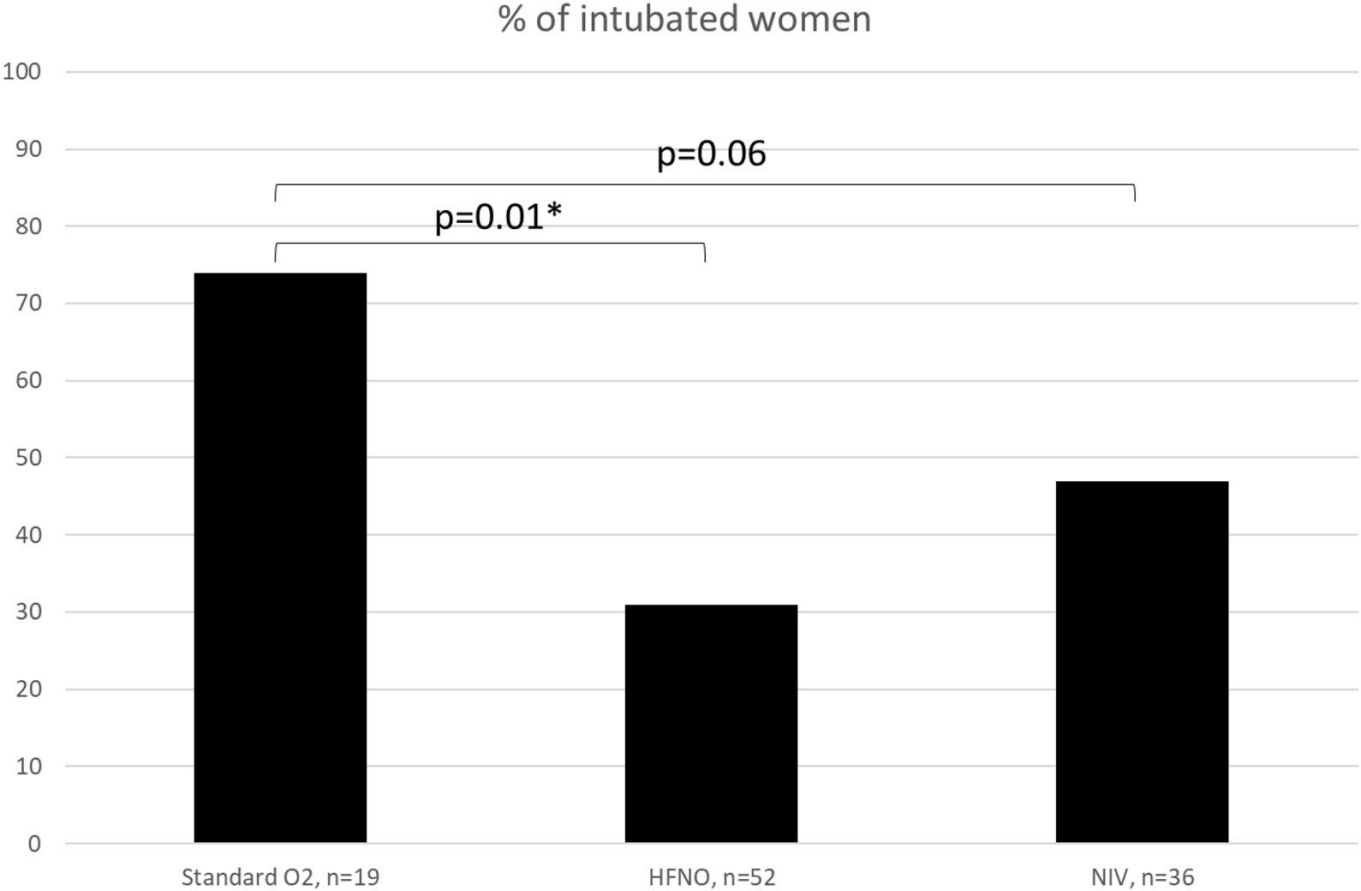
Women requiring intubation according to respiratory support. *HFNO* high-flow nasal oxygen, *NIV* non-invasive mechanical ventilation. Groups of respiratory support were defined by the invasiveness of devices assuming that standard O_2_ is less invasive than HFNO and that HFNO is less invasive than NIV. Patients in whom more than one respiratory device was used were classified in the most invasive group. *Significant p value after Bonferroni correction for multiple comparisons

**Fig. 3:**
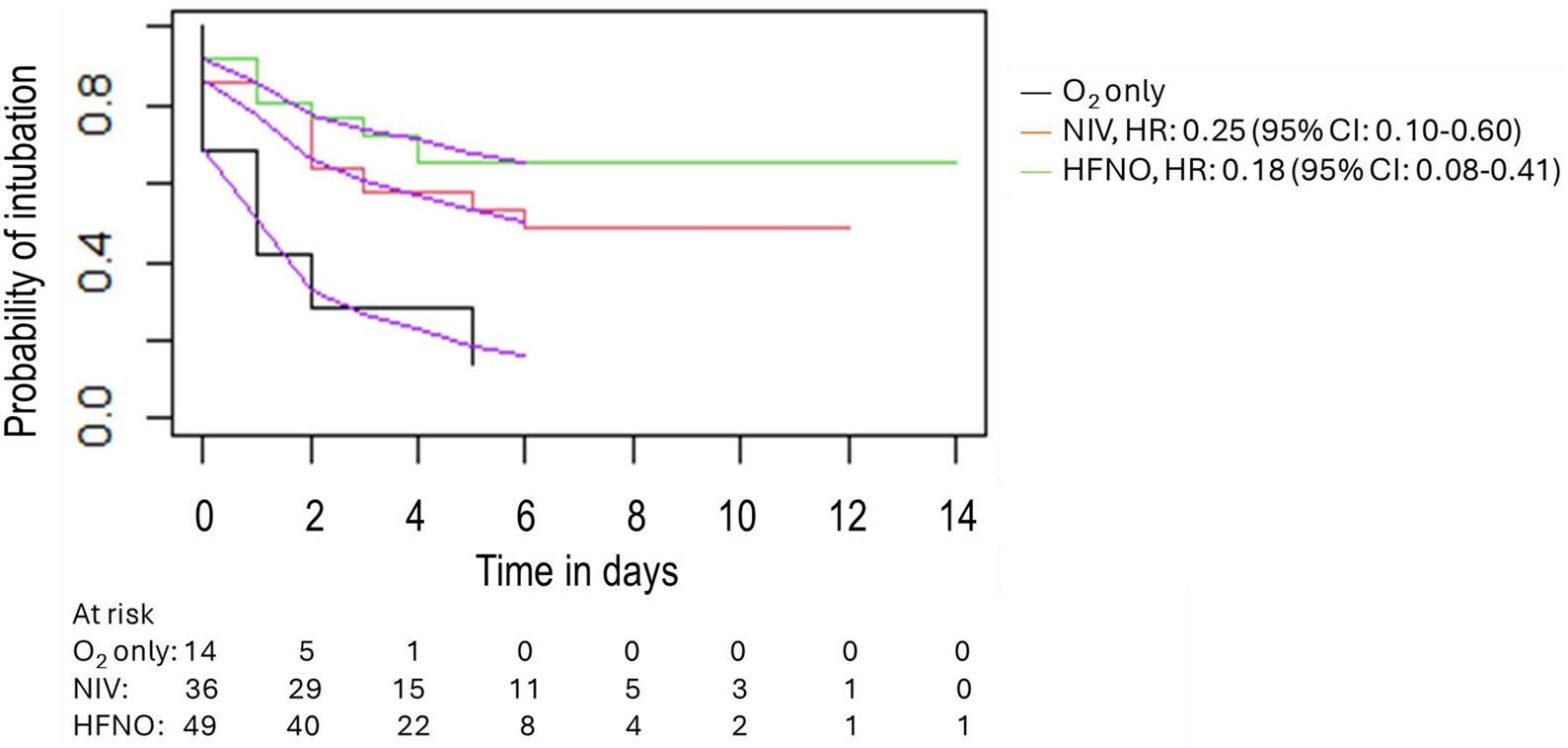
Risk of intubation over time according to the respiratory support Purple lines represent the adjusted risk from the Cox model (n = 99). HR: hazard ratio, *CI* confidence interval, *HFNO* high-flow nasal oxygen, *NIV*   non-invasive mechanical ventilation

Intubation rate was significantly different according to respiratory support (Table 1). After Bonferroni correction, compared to oxygen only, intubation rate was significantly lower in the HFNO group (Fig. 2). Whatever the type of respiratory support, all women were intubated within the first 5 days of ICU admission (Fig. 3). After adjustment, HFNO and NIV were significantly associated with a lower risk of intubation (Fig. 3) and suspected or proven pulmonary co-infection with an increased rate (Table 2). A sensitivity analysis was performed including proven pulmonary co-infection only in the model; the result of the regression was similar (Table S 1).

**Table 2:** Factors associated with intubation in the whole population

	OR (95% CI), p	Adjusted OR (95% CI), p
Proven or suspected pulmonary co-infection	3.23 (1.37–7.60), 0.007	3.39 (1.31–9.21), 0.01
Ventilatory support		
Standard O_2_	1	1
HFNO	0.16 (0.05–0.52), 0.02	0.11 (0.02–0.41), 0.001
NIV	0.32 (0.10–1.07), 0.07	0.20 (0.04–0.80), 0.03
PaO_2_:FiO_2_ ratio < 100 mmHg	3.35 (0.81–6.81), 0.12	3.02 (0.93–10.18), 0.06

Area under the curve: 0.73.

HFNO: high-flow nasal oxygen, NIV: non-invasive ventilation.

### Obstetric management during ICU stay

All women were admitted to the ICU with ongoing pregnancy, and 46 (43%) were delivered during ICU stay (Table 3). The indication for delivery was maternal respiratory worsening in 39/46 (85%). Two women were delivered for foetal distress and one for eclampsia. Among the 46 deliveries in the ICU, 42 (91%) were performed soon (< 2 days) after ICU admission or intubation. Mode of delivery was caesarean section in 42 (91%). The median term at birth was 30 (28–36) weeks (Table 3). Twenty of 46 women were not intubated when the decision to deliver was taken. Fourteen of them were delivered (11 by caesarean section) for maternal respiratory worsening under regional anaesthesia with standard O_2_ in 5 patients, HFNO in 8, and NIV in 1. All 5 women on standard O_2_ and 4/8 on HFNO ultimately required unplanned intubation during or a few hours after delivery, 1/9 woman required vasopressor initiation during delivery and 2/9 required ECMO. The three women delivered by the vaginal route under neuraxial anaesthesia ultimately required intubation. Regional anaesthesia was predominantly performed during the third wave (9/14) and in all 9 cases, except 1, under HFNO/NIV. Twenty-six of the 46 women delivered were on invasive mechanical ventilation when the decision to deliver was taken, 23/26 less than 2 days after intubation.

**Table 3:** Obstetric and neonatal outcomes in the 46 women delivered during ICU stay

	All *N* = 46	Intubated *N* = 39	Not intubated *N* = 7	p
ICU admission after transfer to level 3 maternity hospital, *n* %	11	10 (26)	1 (14)	0.46
Preeclampsia, *n* %	5 (5)	3 (6)	2 (3)	0.65
Indication(s) of delivery, *n* (%):				0.99
Maternal pulmonary worsening	39 (85)	33 (85)	6 (86)	
Foetal distress	2	1	1	
Eclampsia	1	1	0	
Spontaneous labour	2	2	0	
Induction of labour	3	3	0	
Mode of delivery, *n* %				0.99
Caesarean section	42 (91)	35 (89)	7 (100)	
Vaginal delivery	4 (9)	4 (5)	0	
Postpartum haemorrhage (> 500 mL), *n* %	11 (24)	10 (26)	1 (14)	0.46
Gestational age at delivery, weeks	30 (28–36)	29 (27–34)	37 (35–41)	< 0.001
< 28 0/7 weeks, *n* %	12 (26)	12 (31)	0	< 0.001
28 0/7—31 6/7 weeks, *n* %	17 (37)	16 (41)	1 (14)	
32 0/7–35 6/7 weeks, *n* %	12 (26)	8 (20)	4 (57)	
> 35 6/7 weeks, *n* %	5 (11)	3 (8)	2 (29)	
Birthweight, g	1500 (1203–2238)	1443 (1098–1955)	2590 (2088–2789)	0.008
NICU admission, *n* %	29 (63)	26 (67)	3 (42)	0.40
Stillbirth, *n* %	1 (1)	1 (2)	0	0.99

*NICU* neonatal intensive care unit

We identified 4 different trajectories in managing delivery depicted in Table 4 and figure S3:

1. Nineteen women at a median gestational age of 34 (29–36) weeks were delivered within 2 days after ICU admission while not intubated. Respiratory support when the decision to deliver was taken was standard O_2_ in 10, HFNO in 8, and NIV in 1. Finally, 12/19 (63%) required invasive mechanical ventilation; 3 among them required ECMO.
2. Twenty-three women at a median gestational age of 28 (27–30) weeks were delivered within 2 days of intubation.
3. In 11 women at a gestational age of 24 (23–28) weeks, pregnancy was continued more than two days after intubation. Two were delivered after 3 and 17 days of invasive mechanical ventilation and one stillbirth was recorded at a term of 28 weeks while the patient was on ECMO. The remaining 8 patients were delivered at full term after ICU discharge.
4. Fifty-four women at a gestational age of 27 (24–28) weeks were not delivered soon after ICU admission and never required intubation. Four were managed with standard O_2_, 32 with HFNO for 3 (IQR: 2–5) days and 18 received NIV for 3 (IQR: 1–5) days. All except one were delivered after ICU discharge.

**Table 4:** Comparison of patient characteristics between delivery strategies

	Early delivery while not intubated YES, *n* = 19	Delayed delivery never intubated *N* = 54	Early delivery after intubation *N* = 23	Delayed delivery after intubation *N* = 11	P**
ICU admission after transfer to a level 3 maternity hospital, *n* (%)	2 (11)	12 (22)	7 (30)	5 (45)	0.28
Age, y	34 (31–36)	33 (30–39)	35 (32–38)	33 (28–39)	0.76
BMI	26 (21–31)	28 (25–32)	29 (25–31)	29 (24–34)	0.34
Pneumonia characteristics					
Time from 1st symptoms to admission, d	6 (4–8)	7 (5–8)	7 (5–9)	4 (3–7)	0.11
CT scan, *n* %	17 (89)	45 (83)	18 (78)	7 (64)	0.44
Extent > = 50% on CT-scan, *n* %	4 (25)	15 (33)	7 (39)	1 (9)	0.78
Pulmonary co-infection, *n* %	8 (42)	10 (18)	10 (43)	5 (45)	0.04
Steroids started before ICU admission, *n* %	10 (53)	29 (54)	6 (26)	6 (55)	0.14
Obstetric characteristics					
Gestational age on admission, w	34 (29–36)	27 (24–28)	28 (27–29)	24 (23–28)	< 0.01
Admission < 28 weeks, n %	2 (11)	35 (65)	8 (35)	7 (63)	< 0.01
Respiratory parameters					
PaO_2_:FiO_2_ ratio mmHg	160 (133–197)	177 (139–200)	144 (90–221)	146 (130–190)	0.56
PaO_2_:FiO_2_ < 100 mmHg, *n* %	1 (5)	0	6 (25)	0	< 0.01
pH	7.37 (7.28–7.42)	7.44 (7.40–7.47)	7.45 (7.42–7.46)	7.43 (7.41–7.45)	0.013
PaCO_2_, mmHg	32 (20–36)	32 (30–34)	31 (26–33)	34 (30–40)	0.59
Respiratory rate, b/min	30 (28–37)	31 (25–36)	35 (26–38)	28 (26–39)	0.83
Respiratory support, *n* %					< 0.01
Standard O_2_	10 (53)	4 (7)	4 (17)	1 (10)	
HFNO	6 (31)	32 (59)	9 (39)	5 (45)	
NIV	3 (16)	18 (34)	10 (44)	5 (45)	
Maternal outcome in the ICU					
Requiring ECMO, *n* %	3 (16)	–	5 (21)	2 (2)	*
Death	0	0	1	0	*
Thromboembolic event, *n* %	2	1	3	1	0.11
Obstetric outcome in the ICU					
Delivery during ICU stay, *n* %	19 (100)	1 (19)	23 (100)	3 (27)	*
Indication of delivery, *n* %					*
Respiratory maternal worsening	14 (74)	1 (100)	23 (100)	1 (33)	
Foetal distress	1 (5)	0	0	1 (33)	
Stillbirth	0	0	0	1 (9)	NA
Term of birth					< 0.01
28 0/7 weeks, *n* %	3 (16)	0	7 (30)	2 (67)	
28 0/7—31 6/7 weeks, *n* %	3 (16)	0	13 (56)	1 (33)	
32 0/7–35 6/7 weeks, *n* %	8 (42)	1 (100)	3 (14)	0	
> 35 6/7 weeks, *n* %	5 (26)	0	0	0	

*NA* not applicable

*HFNO* high-flow nasal oxygen, *NIV* non-invasive ventilation, *ECMO* extracorporeal membrane oxygenation

*Not analyzed

**4 group comparisons by the Kruskal–Wallis test

The main difference between the 4 strategies was gestational age on admission (Table 4). Early delivery after intubation resulted in the most frequent prematurity (Table 4). All six intubated women with severe hypoxaemia characterized by a PaO_2_:FiO_2_ ratio < 100 mmHg were early delivered after intubation. Three were rapidly extubated after 2 days, 1 after 6 days, 1 after 3 weeks and 1 required ECMO.

### Maternal outcomes

One maternal death was recorded in a patient managed with ECMO started after delivery (figure S3). Complications related to ICU stay were more frequent in intubated patients (Table 5). Nosocomial infection was observed in 29 women, 24 of them after intubation. Nineteen experienced ventilator-associated pneumonia and three catheter-related bloodstream infections. Documented thromboembolic events occurred in 7 patients during ICU stay, all except one in intubated patients.

**Table 5:** Maternal outcome of the 107 women included

	All *N* = 107	Intubated *N* = 47	Not intubated *N* = 60	*p*
Vasopressors, *n* %	28 (26)	28 (60)	0	< 0.001
Before delivery	11 (10)	11 (23)		
After delivery	17 (16)	17 (36)		
Cardiac arrest, *n* %	1	1	0	0.43
Before delivery	0	0		
After delivery	1	1		
Barotrauma, *n* %	4 (4)	4 (9)	0	0.035
Before delivery	0	0		
After delivery	4 (4)	4 (9)		
Prone positioning, *n* %	29 (27)	25 (53)	4 (7)	< 0.001
Started before delivery	9 (9)	6 (13)	3 (5)	
ECMO, *n* %	10 (9)	10 (21)	–	
Nosocomial infection (at least one), *n* %	29 (27)	24 (51)	5 (8)	< 0.001
Ventilator-associated pneumonia, *n* %	19 (18)	19 (40)	NA	NA
Documented thromboembolic complications, *n* %				
All	7 (7)	6 (13)	1 (2)	0.042
Pulmonary embolism	4 (4)	3 (6)	1 (2)	
Deep venous thrombosis	3 (3)	3 (6)	0	
Renal replacement therapy, *n* %	2 (2)	2 (4)	0	0.19
ICU length of stay, d	6 (3–11)	11 (6–26)	4 (3–6)	< 0.001
Hospital length of stay, d	15 (11–23)	25 (14–48)	13 (11–16)	< 0.001
Hospital death, *n* %	1 (1)	1 (2)	0	0.99

*NA* not analyzed

*ECMO* extracorporeal membrane oxygenation, all were veno-venous

### Neonatal outcomes

One stillbirth was recorded in a patient on ECMO. Among the 45 infants born during the mother’s ICU stay, 12 (29%) were extremely, 17 (40%) very and 12 (27%) moderately preterm (Table 3). Median birth weight of the 45 infants was 1500 (1203–2238) g. Neonatal ICU admission was required in 29/45 (64%) infants. Ninety-four women were admitted at a term lower than 34 weeks, 53 received steroids for foetal lung maturation (45 betamethasone and 8 dexamethasone), and steroids were continued in 41/53 for COVID treatment. Nineteen women were delivered in the ICU before 32 weeks, all having received steroids for lung maturation.

## Discussion

In this large cohort of pregnant women with AHRF related to SARS-CoV-2 infection, we found that both HFNO and NIV used in 48% and 34% of pregnant women, respectively, are associated with a lower rate of intubation compared to standard O_2_. Our results are in accordance with previous reports on oxygen support in other populations of COVID patients not including pregnant women [15, 16]. It has been demonstrated that, in patients with AHRF related to COVID, HFNO significantly reduced intubation rate from 55 to 45%, but did not change mortality [15]. Recent European guidelines on AHRF management strongly recommend HFNO use to reduce the risk of intubation, whatever the cause of lung injury [17]. Whether NIV can be considered is less certain; however, in AHRF due to COVID‑19, these European guidelines suggest that NIV, at one (continuous positive airway pressure) or two levels (bi-level positive airway pressure) of pressure, can be considered instead of HFNO to reduce intubation. Different practices of non-invasive respiratory support have been reported in obstetric patients with COVID. HFNO and NIV, respectively, were used in 64% and 22% of cases in European countries [9], 40% and 2% in South America [10] and 7% and 0% in Israel [18]. In a US multicentre study, 67% of antenatal pregnant women received NIV/HFNO [19].

We found discrepant results regarding factors associated with intubation compared to previous reports of obstetric patients with COVID. In a prospective study performed in 91 patients managed in South American ICUs, intubation rate was not significantly different using standard O_2_ or HFNO (66% vs 75%) [10]. Intubation was particularly frequent in this study (76%) and analysis regarding NIV was not possible as there were only 2 women in this group. In a retrospective European study including 187 ante- or postnatal women with COVID referred to the ICU, obesity was associated with an increased risk of intubation, whereas it was not in our cohort [9]. In this same European study, intubation rates were 42% and 56% in women managed with HFNO or NIV, respectively, and zero in women managed with standard O_2_. After adjustment, compared to standard O_2_, NIV use was associated with an increased rate of intubation and HFNO had no effect. Some differences from our study can explain these different results. Firstly, none of the women managed with standard O_2_ required intubation. In our study and that of Vasquez et al. [10], the intubation rate in women managed with standard O_2_ was 74% and 75%, respectively. In our study, patients under oxygen received at least 6 L/min. Secondly, the risk of intubation was analysed in a mixed population of ante- and postnatal women, while all women were prenatal in our study. Thirdly, in our study, NIV was most frequently used as first-line respiratory support. A higher rate of intubation found with NIV in other studies could be related to different practices in using NIV as rescue therapy just before intubation. Fourthly, intubation criteria differ between countries and centres and this could explain the different results and also limits the external validity of our results. As reported in figure S2, intubation rate varied between centres, the number of women included per centre was too limited to take into account centre effects in the analysis. The effect of NIV shown in our study should be interpreted bearing in mind that it was combined with HFNO in almost all women. This information is not available in previous studies.

For the first time, we report the number of bacterial pulmonary co-infections in pregnant women with COVID. Co-infection was a factor strongly associated with an increased rate of intubation, even when considering proven infection only in our sensitivity analysis. Greater severity in women experiencing co-infection is illustrated by the higher number who required ECMO after intubation: 7/21 (33%) compared to 3/26 (12%) in patients without co-infection. Two French cohort studies in COVID patients, in general, reported 19% and 28% of proven co-infections at ICU admission [20, 21]. When counting documented infections only, we found that 16% of women experienced co-infections. As previously reported in France, *Staphylococcus aureus* was most frequently isolated [20, 21]. The low incidence of bacterial co-infection found in our study does not support systematic empirical use of antibiotics in pregnant women with COVID.

Guidelines recommend early prone positioning in patients intubated for severe-moderate ARDS to reduce mortality and suggest the use of awake prone positioning in COVID patients to reduce intubation [17]. Prone positioning during pregnancy is a technical challenge in ensuring good positioning without uterus compression and may affect foetal monitoring. Twenty-nine patients were managed with prone positioning, before delivery in 9 (31%) of them. In a Dutch case series of obstetric patients with COVID, when prone positioning was applied it was started before delivery in 70% of women [22], compared with 37% in the European cohort [9] and 69% in the South American cohort [10]. In the latter, because no serious adverse effect was reported, the authors indicate that their results may encourage physicians to use prone positioning in ongoing pregnancy. This conclusion should, however, be viewed in light of the high maternal (18%) and foetal (15%) mortality reported in this study.

Regarding obstetrical management, the decision when to deliver was justified by maternal pulmonary worsening in most intubated and non-intubated women. For the first time, we report the feasibility of neuraxial anaesthesia in a context of AHRF. Although the primary aim of choosing regional anaesthesia is to avoid intubation, the majority of women were ultimately intubated. The question of whether neuraxial anaesthesia contributed to the worsening of pulmonary function, by itself, remains open. Lumbar epidural anaesthesia is not expected to worsen respiratory function by respiratory muscle blockade. Most of these women were delivered by caesarean section, so that an increase in oxygen consumption related to labour was prevented. Supine positioning for delivery with insufficient positive expiratory pressure could had been deleterious for respiratory mechanics [23]. All women were delivered for pulmonary worsening and, therefore, they could have been finely intubated because of the natural progression of their disease. Neuraxial anaesthesia was mostly performed during the third wave and under HFNO/NIV, suggesting that physicians were probably more confident with these respiratory supports and less reluctant to choose regional anaesthesia despite the presence of respiratory failure. This underlines that non-invasive oxygenation techniques must be used in validated indications.

The question of continuing a pregnancy on invasive mechanical ventilation is central to the right timing of delivery. Not surprisingly, our results suggest that the timing of delivery was mainly dictated by gestational age. Women not delivered soon after ICU admission or intubation were predominantly at a term lower than 28 weeks. Findings from other groups show that, in women with COVID, pregnancy was continued on invasive mechanical ventilation in 23% [9], 21% [18] and 9% [10] of intubated women. The higher percentage (i.e. 32%) observed in our study can be explained by the lower gestational age of the women included. Continuing pregnancy in intubated women at an early gestational age could also be less complex because of better parietal compliance and easier prone positioning. Severe hypoxaemia also seems to have been a trigger for early delivery after intubation. The six women included with a PaO_2_:FiO_2_ ratio less than 100 mmHg were all delivered early after intubation. Interestingly, three of them were rapidly extubated despite severe prenatal hypoxaemia, suggesting a possible improvement of respiratory function after delivery. Early delivery after intubation could also be justified by the need for ECMO. Eight of the 10 women needing ECMO were delivered before the procedure. In the reported experience of French centres in managing ECMO in 24 pregnant women suffering from pulmonary viral infection (13 related to SARS-CoV-2), while maternal survival was similar when ECMO was started before (n = 11/24) or after (n = 13/24) delivery, foetal outcome appeared worse when started before delivery, with only 6/11 alive at birth [24].

Belief in respiratory improvement following delivery needs to be tempered in regard to the weakness of data reported in the literature and conflicting results. We observed that early delivery after ICU referral did not avoid intubation and prolonged invasive ventilation in three-quarters of women. Péju and colleagues reported the respiratory parameters of 11 pregnant women with COVID delivered on invasive mechanical ventilation [9]. Because of the retrospective design, data were not recorded at similar time points. The main result was strong inter-individual variability in the outcome, which precludes any firm conclusion. This raises the question of possible predictive factors for respiratory improvement after delivery. The largest prospective assessment of the evolution of respiratory function after delivery was performed by Vasquez et al. in 47 intubated pregnant women with COVID, one-quarter of whom had a PaO_2_:FiO_2_ below 100 mmHg [10]. Driving pressure, plateau pressure and compliance did not change significantly after delivery, while PaO_2_:FiO_2_ ratio improved significantly 2 and 24 h after. Although oxygenation improvement is not predictive of a better outcome in ARDS patients, delivery could be regarded as a rescue therapy that improves hypoxaemia in patients unable to maintain reasonable oxygenation, particularly when pregnancy complicates the decision to implement prone positioning or ECMO.

Compared to the mortality rate of 31% reported in COVID patients in general referred to French ICUs during the first wave, survival was better in pregnant women with COVID [25]. However, the mortality of 1% observed in our study should rather be compared to the mortality of 3% reported in patients of similar age to pregnant women (i.e. less than 40 years) and referred to French ICUs for COVID during the same period (i.e. third wave) [26]. Maternal ICU complications were significantly higher when patients were intubated, with nosocomial infection being the most frequent. Although pregnant women are at increased risk of thrombosis, thromboembolic events were documented in only 7% of patients in our study. Note that the large majority of women included received reinforced preventive anticoagulation (Fig. 1). In other cohorts of obstetric patients with COVID, thrombotic events acquired in the ICU occurred in 2 to 13% of cases [9, 10, 19], compared with 15% reported in the general population of ICU patients with COVID [27]. Steroid use increased over time and dexamethasone was used in the majority of women according to the RECOVERY study [28]. However, in the absence of indication for foetal lung maturation, other steroids not crossing the placenta should be preferred.

Our study provides additional information in an undervalued field of critical care. Centres and inclusion criteria were chosen to allow the selection of pregnant women with AHRF at an early gestational age in whom the decision when to deliver is trickiest. The main strength of our study is the reported experience from expert centres faced with similar pressure of COVID admissions. Unlike previous studies, all women included had ongoing pregnancies and, therefore, constituted a more homogeneous population for the analysis of risk factors of intubation. Despite the retrospective design of our study, we were able to collect the timings of events needed for trajectory classification.

We acknowledge that our study has several limitations. In the absence of consensual criteria, the decision to intubate is subjective and varies between centres and physicians. Availability of ICU beds may also have influenced the decision. The number of missing data for arterial blood gases was too high to be included in the model of risk factors for intubation. Our results should be interpreted with regard to changing practices and growing experience over the study period and cannot be extrapolated to the more recent omicron variant or to a vaccinated population of pregnant women. A large majority of deliveries were justified by pulmonary worsening in the mother without the possibility to document worsening objectively. However, ICU admission and intubation were frequent triggers that per se are markers of worsening. Lastly, the low foetal death rate can be explained by the high number of inborn infants. The outcome of infants born to women delivered during ICU stay in a non-tertiary centre could be worse. Despite the low foetal death rate observed in our study, later infant outcome needs to be assessed.

## Conclusion

HFNO and NIV were associated with a lower rate of intubation, suggesting that both can be considered in pregnant women with AHRF related to COVID. Over half of the women delivered under regional anaesthesia ultimately required unplanned intubation. Almost all deliveries during ICU stay were justified by maternal pulmonary worsening and performed soon after ICU admission or intubation. Despite good maternal and neonatal survival, delivery in the ICU led to frequent prematurity, particularly in intubated women. Pregnancy was continued on invasive ventilation in only one-third of women.

### Supplementary Information

Supplementary material 1: Figure S1: Flow chart. Figure S2: Inclusions by centre over time. Figure S3: Maternal trajectories for delivery strategy. Table S1: Sensitivity analysis of intubation risk factors including only proven pulmonary co-infections.

## Data Availability

The data sets used and/or analysed during the current study are available from the corresponding author on reasonable request.

## Appendix

See Table 6

**Table 6:** STROBE Statement

	Item No	Recommendation	Page
Title and abstract	1	(*a*) Indicate the study’s design with a commonly used term in the title or the abstract	1
		(*b*) Provide in the abstract an informative and balanced summary of what was done and what was found	5
Introduction			
Background/rationale	2	Explain the scientific background and rationale for the investigation being reported	6
Objectives	3	State specific objectives, including any prespecified hypotheses	6
Methods			
Study design	4	Present key elements of study design early in the paper	7
Setting	5	Describe the setting, locations, and relevant dates, including periods of recruitment, exposure, follow-up, and data collection	7
Participants	6	(*a*) Give the eligibility criteria, and the sources and methods of selection of participants. Describe methods of follow-up	7
		(*b*) For matched studies, give matching criteria and number of exposed and unexposed	NA
Variables	7	Clearly define all outcomes, exposures, predictors, potential confounders, and effect modifiers. Give diagnostic criteria, if applicable	8
Data sources/ measurement	8	For each variable of interest, give sources of data and details of methods of assessment (measurement). Describe comparability of assessment methods if there is more than one group	*7*
Bias	9	Describe any efforts to address potential sources of bias	9
Study size	10	Explain how the study size was arrived at	NA
Quantitative variables	11	Explain how quantitative variables were handled in the analyses. If applicable, describe which groupings were chosen and why	8
Statistical methods	12	(*a*) Describe all statistical methods, including those used to control for confounding	8
		(*b*) Describe any methods used to examine subgroups and interactions	8
		(*c*) Explain how missing data were addressed	9
		(*d*) If applicable, explain how loss to follow-up was addressed	NA
		(*e*) Describe any sensitivity analyses	11
Results			
Participants	13	(a) Report numbers of individuals at each stage of study—eg numbers potentially eligible, examined for eligibility, confirmed eligible, included in the study, completing follow-up, and analysed	Figure S1
		(b) Give reasons for non-participation at each stage	Figure S1
		(c) Consider use of a flow diagram	Figure S1
Descriptive data	14	(a) Give characteristics of study participants (eg demographic, clinical, social) and information on exposures and potential confounders	Table 1 and page 9
		(b) Indicate number of participants with missing data for each variable of interest	Table 1
		(c) Summarise follow-up time (eg, average and total amount)	NA
Outcome data	15	Report numbers of outcome events or summary measures over time	Figure 3
Main results	16	(*a*) Give unadjusted estimates and, if applicable, confounder-adjusted estimates and their precision (eg, 95% confidence interval). Make clear which confounders were adjusted for and why they were included	Tables 1, 2, Fig. 3
		(*b*) Report category boundaries when continuous variables were categorized	NA
		(*c*) If relevant, consider translating estimates of relative risk into absolute risk for a meaningful time period	NA
Other analyses	17	Report other analyses done—eg analyses of subgroups and interactions, and sensitivity analyses	Table S1
Discussion			
Key results	18	Summarise key results with reference to study objectives	Discussion section
Limitations	19	Discuss limitations of the study, taking into account sources of potential bias or imprecision. Discuss both direction and magnitude of any potential bias	17
Interpretation	20	Give a cautious overall interpretation of results considering objectives, limitations, multiplicity of analyses, results from similar studies, and other relevant evidence	Discussion section
Generalisability	21	Discuss the generalisability (external validity) of the study results	14
Other information			
Funding	22	Give the source of funding and the role of the funders for the present study and, if applicable, for the original study on which the present article is based	3

## Abbreviations

AHRF: Acute hypoxaemic respiratory failure
ECMO: Extracorporeal membrane oxygenation
HFNO: High-flow nasal oxygen
ICU: Intensive care unit
NICU: Neonatal intensive care unit
NIV: Non-invasive mechanical ventilation
