# Supplementary material for: Management of pregnant women in tertiary maternity hospitals in the Paris area referred to the intensive care unit for acute hypoxaemic respiratory failure related to SARS-CoV-2: which practices for which outcomes?
Source: Ann Intensive Care. 2024 Jun 18;14:94. doi: 10.1186/s13613-024-01313-2 (PMC11189363; doi:10.1186/s13613-024-01313-2)
Supplement: Supplementary file 1 — Supplementary material 1: Figure S1: Flow chart. Figure S2: Inclusions by centre over time. Figure S3: Maternal trajectories for delivery strategy. Table S1: Sensitivity analysis of intubation risk factors including only proven pulmonary co-infections. [file 13613_2024_1313_MOESM1_ESM.docx]

**Electronic Supplementary Material**

Figure S1: Flow chart

Figure S2: Inclusions by centre over time

Figure S3: Maternal trajectories for delivery strategy

Table S1: Sensitivity analysis of intubation risk factors including only proven pulmonary co-infections

Appendix: STORBE statement

**Figure S1: Flow chart**

**
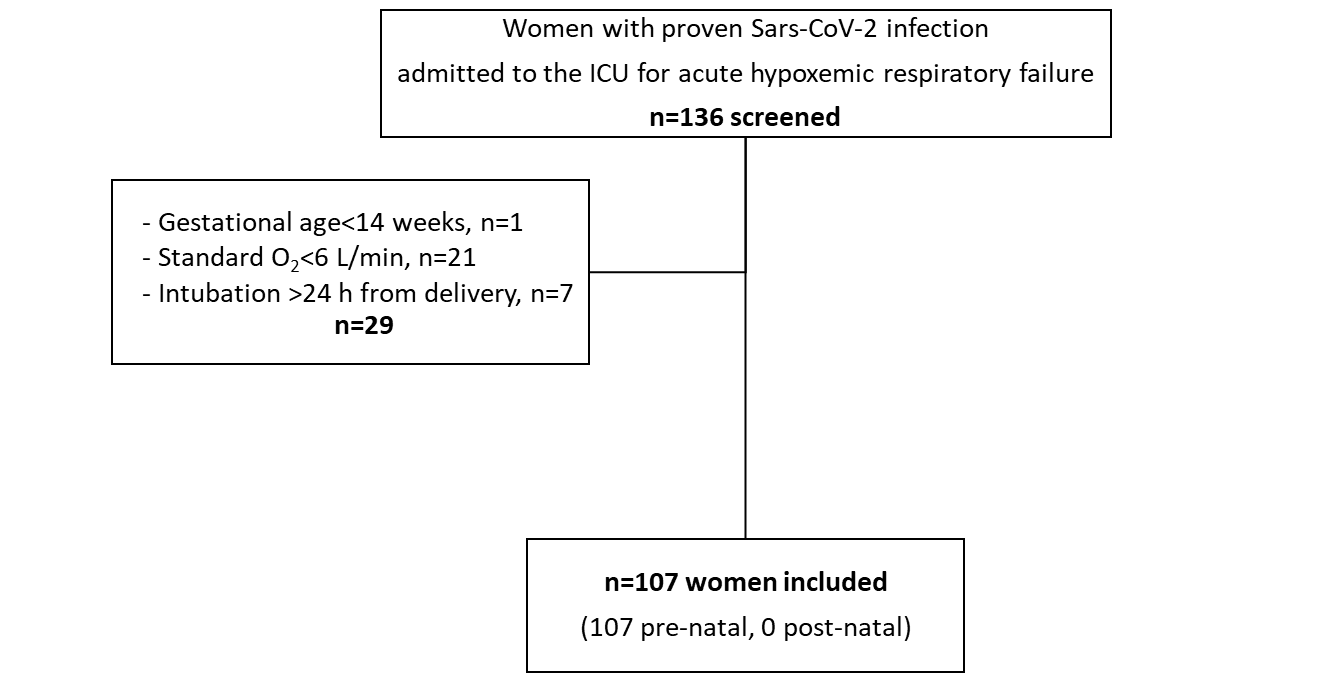
**

**Figure S2: Inclusion by centre over time**

**Panel A: Inclusions over time**

**Panel B: inclusion by centre**

**
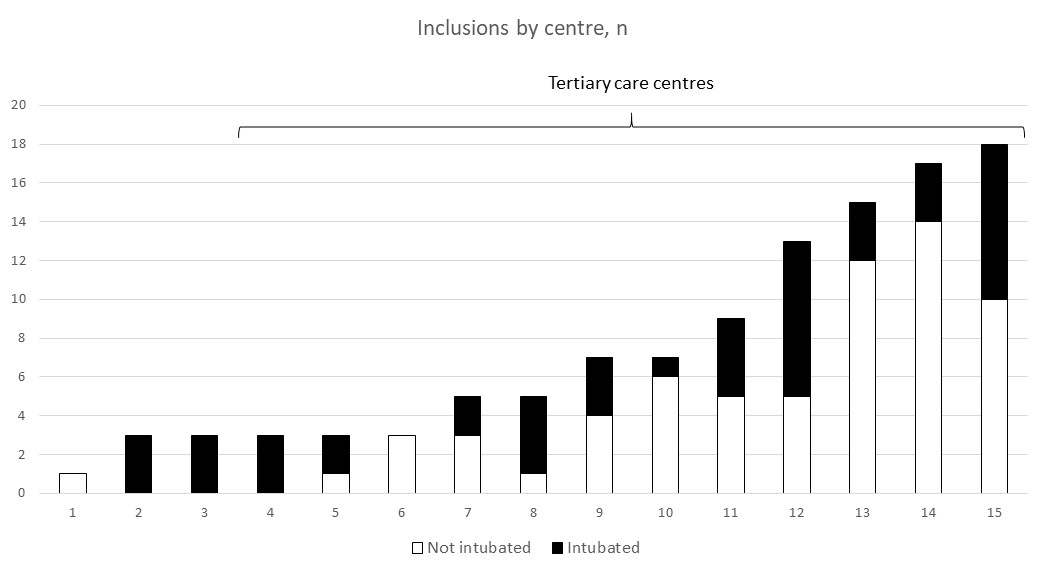
**

**Figure S3: Maternal trajectories for delivery strategy**

Days

N= non-invasive ventilation, H= high-flow nasal oxygen, P= prone position, MD= maternal death, SB= stillbirth

**Figure S3 continued**

CORRECTION

Not early delivered => Not delivered early

**Table S1: Sensitivity analysis of intubation risk factors including only proven pulmonary co-infections**

|  | **Adjusted OR (95% CI), p** |
| --- | --- |
| Proven pulmonary co-infection | 5.39 (1.26-22.88), 0.022 |
| Ventilatory support:  Standard O_2_  HFNO  NIV | 1  0.14 (0.04-0.55), 0.005  0.24 (0.06-0.98), 0.047 |
| PaO_2_:FiO_2_ ratio <100 mmHg | 10.24 (1.06-98.96), 0.06 |

**Appendix**

|  | Item No | Recommendation | Page |
| --- | --- | --- | --- |
| **Title and abstract** | 1 | (*a*) Indicate the study’s design with a commonly used term in the title or the abstract | 1 |
|  |  | (*b*) Provide in the abstract an informative and balanced summary of what was done and what was found | 5 |
| Introduction | | |  |
| Background/rationale | 2 | Explain the scientific background and rationale for the investigation being reported | 6 |
| Objectives | 3 | State specific objectives, including any prespecified hypotheses | 6 |
| Methods | | |  |
| Study design | 4 | Present key elements of study design early in the paper | 7 |
| Setting | 5 | Describe the setting, locations, and relevant dates, including periods of recruitment, exposure, follow-up, and data collection | 7 |
| Participants | 6 | (*a*) Give the eligibility criteria, and the sources and methods of selection of participants. Describe methods of follow-up | 7 |
|  |  | (*b*) For matched studies, give matching criteria and number of exposed and unexposed | NA |
| Variables | 7 | Clearly define all outcomes, exposures, predictors, potential confounders, and effect modifiers. Give diagnostic criteria, if applicable | 8 |
| Data sources/ measurement | 8* | For each variable of interest, give sources of data and details of methods of assessment (measurement). Describe comparability of assessment methods if there is more than one group | *7* |
| Bias | 9 | Describe any efforts to address potential sources of bias | 9 |
| Study size | 10 | Explain how the study size was arrived at | NA |
| Quantitative variables | 11 | Explain how quantitative variables were handled in the analyses. If applicable, describe which groupings were chosen and why | 8 |
| Statistical methods | 12 | (*a*) Describe all statistical methods, including those used to control for confounding | 8 |
|  |  | (*b*) Describe any methods used to examine subgroups and interactions | 8 |
|  |  | (*c*) Explain how missing data were addressed | 9 |
|  |  | (*d*) If applicable, explain how loss to follow-up was addressed | NA |
|  |  | (*e*) Describe any sensitivity analyses | 11 |
| Results | | |  |
| Participants | 13* | (a) Report numbers of individuals at each stage of study—eg numbers potentially eligible, examined for eligibility, confirmed eligible, included in the study, completing follow-up, and analysed | Figure S1 |
|  |  | (b) Give reasons for non-participation at each stage | Figure S1 |
|  |  | (c) Consider use of a flow diagram | Figure S1 |
| Descriptive data | 14* | (a) Give characteristics of study participants (eg demographic, clinical, social) and information on exposures and potential confounders | Table 1 and page 9 |
|  |  | (b) Indicate number of participants with missing data for each variable of interest | Table 1 |
|  |  | (c) Summarise follow-up time (eg, average and total amount) | NA |
| Outcome data | 15* | Report numbers of outcome events or summary measures over time | Figure 3 |
| Main results | 16 | (*a*) Give unadjusted estimates and, if applicable, confounder-adjusted estimates and their precision (eg, 95% confidence interval). Make clear which confounders were adjusted for and why they were included | Table 1, table 2, figure 3 |
|  |  | (*b*) Report category boundaries when continuous variables were categorized | NA |
|  |  | (*c*) If relevant, consider translating estimates of relative risk into absolute risk for a meaningful time period | NA |
| Other analyses | 17 | Report other analyses done—eg analyses of subgroups and interactions, and sensitivity analyses | Table S1 |
| Discussion | | |  |
| Key results | 18 | Summarise key results with reference to study objectives | Discussion section |
| Limitations | 19 | Discuss limitations of the study, taking into account sources of potential bias or imprecision. Discuss both direction and magnitude of any potential bias | 17 |
| Interpretation | 20 | Give a cautious overall interpretation of results considering objectives, limitations, multiplicity of analyses, results from similar studies, and other relevant evidence | Discussion section |
| Generalisability | 21 | Discuss the generalisability (external validity) of the study results | 14 |
| Other information | | |  |
| Funding | 22 | Give the source of funding and the role of the funders for the present study and, if applicable, for the original study on which the present article is based | 3 |
